# Prediction of metastatic risk of renal clear cell carcinoma based on CT radiomics analysis

**DOI:** 10.3389/fonc.2025.1576956

**Published:** 2025-06-06

**Authors:** Xueyi Wang, Youchang Yang, Jiaojiao Wu, Xiaoqiang Tang, Yao Wang

**Affiliations:** ^1^ Department of Radiology, Wujin Hospital Affiliated with Jiangsu University 2 The Wujin Clinical college of Xuzhou Medical University, Changzhou, China; ^2^ Department of Radiology, Qilu Hospital (Qingdao), Cheeloo College of Medicine, Shandong University, Qingdao, China; ^3^ Department of Research and Development, Shanghai United Imaging Intelligence Co., Ltd., Shanghai, China; ^4^ Department of Radiology, The Affiliated Changzhou No. 2 People’s Hospital of Nanjing Medical University, Changzhou, China

**Keywords:** clear cell renal cell carcinoma, CT, predicted, radiomics, metastasis

## Abstract

**Objective:**

To investigate the value of using imaging histological models to non-invasively assess the risk of metastasis in patients with clear cell renal cell carcinoma (ccRCC).

**Methods:**

This study retrospectively enrolled 273 clear cell renal cell carcinoma (ccRCC) patients from three hospitals, with 57 cases allocated as an independent test cohort. High-throughput imaging histomic features (n=2,264) were extracted from triphasic CT (non-enhanced, corticomedullary, and nephrographic phases) using Pyradiomics. Three monophasic radiomics models were developed following dimensionality reduction, with feature contributions quantified via Shapley Additive exPlanations (SHAP) framework to enhance interpretability. A triphasic radiomics model was subsequently established by ensembling phase-specific prediction probabilities. Metastasis risk factors identified through univariate/multivariate logistic regression informed a clinical predictor model. The final combined model integrated triphasic radiomics signatures with clinical parameters, visualized through a nomogram. Diagnostic performance was evaluated via ROC analysis, while calibration curves validated prediction consistency.

**Results:**

In this study, SHAP analysis revealed that radiomics features quantifying intratumoral heterogeneity (e.g., necrosis patterns in medullary-phase CT) synergized with clinical factors (tumor size >3 cm, creatinine levels) to drive predictions. Key biological insights included threshold effects of necrosis volume (linked to hypoxia) and tumor diameter (critical threshold: 3 cm), aligning with known metastatic pathways. The clinical model achieved an area under the ROC curve (AUROC) of 0.752 (95% confidence interval [CI]: 0.679-0.826) in the training dataset and 0.681 (95% CI: 0.529-0.833) in the testing dataset. Among the single-phase radiomics models, the CT_Medullary model demonstrated good prediction performance, with an AUROC of 0.785 (95% CI: 0.645-0.924) in the testing dataset. The three-phased CT model exhibited improved diagnostic performance, with a testing AUROC rising to 0.812 (95% CI: 0.680-0.943). Notably, the combined model integrating clinical and radiomics features yielded the best prediction, achieving a further improvement in testing AUROC to 0.824 (95% CI: 0.704-0.944).

**Conclusion:**

Radiomics technology provides a quantitative, objective method for predicting the risk of metastasis in patients with ccRCC. Nonetheless, the clinical indicators persist as irreplaceable.

## 1 Introduction

Renal cell carcinoma (RCC) accounts for approximately 90% of renal malignancies, with clear cell renal cell carcinoma (ccRCC) being the most common subtype (1, 2). Surgery is the most effective radical treatment, but studies have shown that approximately 30% of ccRCC patients present with local recurrence or metastasis at initial presentation (3, 4). ccRCC does not respond well to radiotherapy and chemotherapy, and the 5-year survival rate for patients with metastatic ccRCC is only 10% (5). Therefore, accurate assessment of the risk of ccRCC recurrence and metastasis after surgery is extremely important for patient prognosis. Currently, the prognosis of patients with RCC is mainly predicted by tumor size, TNM staging system, Fuhrman classification, WHO/ISUP classification, and other clinicopathological features with limited accuracy, and patients with RCC of the same stage and/or pathological classification often have different prognoses (6). Therefore, new markers are urgently needed to improve the efficacy of predicting ccRCC recurrence and metastasis for accurate and personalized clinical decision-making.

Computed tomography (CT) is a widely used non-invasive imaging modality for tumor staging and assessment of tumor aggressiveness in ccRCC patients (7). Radiomics, a promising and emerging technique, enables the transformation of medical images into vast amounts of image-related features that can be analyzed in model-building algorithms (8–10). To date, radiomics has been successfully applied in several areas of RCC, including prediction of Fuhrman stages and response to therapy in ccRCC and discrimination of RCC subtypes. However, most studies have focused on developing models based solely on texture analysis, neglecting the importance of clinical risk factors and radiological features that could improve predictive performance (11–14). It is worth noting that multimodal data fusion is expected to enhance diagnostic performance, probably because information from different modalities can complement each other and has already shown excellent capabilities in the domain of treatment and prognostic prediction for glioma, ovarian cancer, and breast cancer, among others (15–17).

The purpose of this study was to develop and validate a radiomics nomogram incorporating CT radiological features and clinical factors to predict the risk of metastasis in ccRCC.

## 2 Materials and methods

### 2.1 Patients

The clinical and imaging data of patients diagnosed with ccRCC between April 2013 and March 2021 at Shandong University Qilu Hospital, Jinan Campus (Hospital A), Shandong University Qilu Hospital, Qingdao Campus (Hospital B), and Changzhou No. 2 People’s Hospital (Hospital C) were retrospectively reviewed. Patients were categorized based on the presence or absence of metastasis at 3 years postoperatively. Ethical approval was granted by the institutional review boards of the three hospitals to access their clinical and imaging records for this study. Due to the retrospective nature of the research, written informed consent was not necessary. The inclusion criteria were: (i) pathologically confirmed ccRCC; (ii) a thorough review of patient data, including three-phased CT scans (i.e., non-enhanced, cortical enhanced, and medullary enhanced phases) and laboratory results. The exclusion criteria were: (i) inability to evaluate the patient’s imaging; (ii) incomplete general or laboratory data; and (iii) a history of other malignancies. Ultimately, 273 patients were included in the study, of whom 89 developed metastases.

### 2.2 Image acquisition and preprocessing

All subjects underwent CT enhancement scanning. Patients at Hospital A were examined using up to five different CT helical/spiral scanners, including General Electric Medical Systems, Philips, Siemens, Canon Medical Systems, and a Toshiba 512-row detector; patients at Hospital B were examined using three different CT helical/spiral scanners. These included a 256-slice CT (GE Revolution CT, GE Healthcare, USA), a dual-source CT (SOMATOM Definition, Siemens Healthineers, Germany), and a dual-source CT (SOMATOM Force, Siemens Healthineers, Germany). Hospital C employed Siemens Germany’s Definition Flash CT for the initial examination. The renal examination was conducted using a scanning scope that extended from the upper pole to the lower pole, encompassing the entire kidney. The scanning parameters employed in the three hospitals included in this study are presented in **Supplementary Table 1**. Consequently, the three-phase CT scan images of the non-enhanced, cortical enhanced, and medullary enhanced phases of one patient were evaluated for quality by two radiologists, and any discrepancies were resolved by a senior radiologist with over two decades of diagnostic experience.

The image preprocessing procedures involved the following steps: (1) pixel resampling to a resolution of 1 × 1 mm²; (2) grey-level normalization using the ± 3 sigma method; and (3) grey-level discretization into 64 distinct levels.

### 2.3 Tumor delineation

All CT images in DICOM format were imported into ITK-SNAP v3.6.0 (www.itksnap.org) for annotation of ccRCC lesions, maintaining their original size and resolution. The region of interest (ROI) was delineated by two experienced radiologists (R1 and R2) on cortical enhanced phase CT images. The radiologists jointly reviewed the images to define the three-dimensional (3D) ROI covering the entire lesion. Following this, the non-enhanced phase, medullary enhanced phase, and cortical phase images were aligned using rigid registration to correct for any motion between acquisitions. The ROIs delineated on the cortical enhanced phase images were then mapped to the other two phases and reviewed by a radiologist for further radiomics analysis. The accuracy of the registration was assessed using the Dice similarity coefficient (DSC), which measures the similarity between the registered mask of the moving image (transformed original mask) and the reference segmentation on the fixed image. A DSC value of 0.80 indicated good registration performance.

### 2.4 Radiomics analysis

The radiomics analysis pipeline, encompassing radiomics feature extraction, feature selection, model construction, and performance evaluation, was conducted using the uAI Research Portal (uRP, United Imaging Intelligence) (18). To improve model interpretability, the Shapley additive explanations (SHAP) method was applied by assigning each feature an importance value in the prediction, providing insights into how the model makes decisions.

#### 2.4.1 Feature extraction and selection

For each imaging modality (i.e., non-enhanced CT, cortical enhanced CT, and medullary enhanced CT), each ROI extracted 2,264 radiomic features in compliance with the Image Biomarker Standardization Initiative (IBSI) (19), encompassing first-order statistics, shape-based features, and texture features. Additionally, each participant owned 42 clinical characteristics, including demographic information, biological data, and ccRCC characteristics. To ensure model’s generalizability, feature selection and model construction was conducted on the training dataset and validated on an independent testing dataset. Among the 273 participants, 216 individuals from Hospital A and Hospital B comprised the training dataset, while the remaining 57 participants from Hospital C formed the independent testing dataset.

To select the most valuable radiomics features for constructing three single-phased CT models, feature standardization was initially performed to eliminate the magnitude differences between various features. Only features with intraclass correlation coefficient (ICC) values greater than 0.75 in both intra-observer and inter-observer agreement tests were retained. The feature selection strategy was customized for each model to balance robustness and performance. For the CT_Cortical model, an *F*-test (*P* < 0.05) was first applied to exclude statistically non-significant features, followed by least absolute shrinkage and selection operator (LASSO) regression (α = 0.08) to further reduce multicollinearity. For the CT_Medullary and CT_Non-enhanced models, minimum redundancy maximum relevance (mRMR) was employed to directly optimize feature relevance-redundancy trade-offs, as these phases exhibited stronger inter-feature correlations. For the clinical model, the univariate logistic regression (*P* < 0.05) identified statistically significant predictors, and mRMR refined the subset by removing redundant variables. These methods were applied sequentially (not independently), with the workflow for each model optimized through grid search and cross-validation. In accordance with Harrell’s guideline, the number of selected features should not exceed 10% of the size of the smallest group (the metastasis group) in the training dataset, which is equivalent to 10 EPP (events per candidate predictor parameter) (20). Consequently, the final number of features in each constructed model was limited to fewer than 7. Detailed parameters in feature selection for models are summarized in **Supplementary Table 2**.

#### 2.4.2 Model construction and validation

Based on the selected features, various data preprocessing techniques were employed for feature standardization, such as Z-score scaler, max_abs scaler, L2 normalization, and quantile transformer. To ensure algorithmic diversity and robustness, six machine learning classifiers – random forest (RF), logistic regression (LR), decision tree (DT), Bagging DT, support vector machine (SVM), and partial least squares-discriminant analysis (PLS-DA) – were evaluated. These classifiers were selected to represent distinct computational paradigms, such as tree-based, linear, ensemble methods. Multiple candidate models were generated by combining feature subsets, preprocessing methods, and classifiers. For each classifier, hyperparameters were optimized *via* grid search on the training dataset using 5-fold cross-validation, with the area under the receiver operating characteristic curve (AUROC) as the optimization metric. The final model for each modality was selected based on the highest cross-validated AUROC in the training dataset and subsequently applied to the testing dataset, ensuring strict separation between model development and validation phases to prevent data leakage.

Finally, four single-modality models were constructed based on selected features from their respective modalities: the CT_Corticle model, CT_Medullary model, CT_Non-enhanced model, and clinical model. For instance, RF outperformed other classifiers for CT_Cortical and CT_Medullary models, likely due to its inherent noise robustness and suitability for high-dimensional radiomics data. To investigate whether combining information from three-phase CT images could improve predictions, a multi-phased CT model was created by integrating predicted probabilities from the three single-modality CT models and passing these to another classifier. Additionally, the potential of combining radiomics with clinical information was explored by developing a combined model, which integrated the predicted probabilities from both the multi-phased CT model and the clinical model. Each of the six final models was identified as the optimal configuration for its respective input features, balancing performance and generalizability through iterative parameter tuning (**Supplementary Table 3**).

After selecting the optimized model with superior performance and robustness, the model was applied to the testing dataset to validate its generalizability. The receiver operating characteristic (ROC) curve was first plotted, allowing for the quantitative calculation of the AUROC. Similarly, the precision-recall (PR) curves suitable for unbalanced sample cases, were plotted to visualize the discrimination efficiency, with the calculation of the area under the PR curve (AUPR). To quantitatively assess the consistency between the actual labels and predicted categories, an additional five metrics were calculated from confusion matrices: accuracy, sensitivity, specificity, precision, and F1 score. Additionally, calibration curves were employed to compare the predictive outputs with the actual outcomes. Decision curves were utilized to demonstrate the clinical net benefit of multi-modality models.

#### 2.4.3 Model interpretability with SHAP and nomogram

SHAP (Shapley Additive exPlanations) is a Python library designed to interpret the prediction outcomes of sophisticated machine learning models based on game theory (21). The foundation of SHAP lies in the concept of Shapley values. These values assign an importance score to each feature for a specific prediction, providing a measure of how much each feature contributes to the model’s output, thereby enabling researchers to peek into the “black box” of complex models. The positive or negative SHAP value is a clear indicator of the nature of a feature’s influence on model prediction, where a positive SHAP value represents that the influence of this characteristic on model prediction is promotional, while a negative SHAP value implies that the feature suppresses the prediction. In our study, we harnessed the capabilities of the SHAP library to comprehensively analyze the impact of each factor on the model’s prediction and explore how these features interact.

Alongside SHAP, we applied nomogram to visually display how factors interact and contribute to the model’s prediction, making it easier to understand the model’s decision-making process. The nomogram combines different predictors into a single diagram, in which each variable’s contribution to the prediction is represented by a scale, and by aligning values on these scales, users can estimate the outcome.

### 2.6 Statistical analysis

The Shapiro-Wilk tests were conducted to assess the normal distribution of continuous variables. Continuous variables were expressed as mean ± standard deviation if approximately normally distributed or as median (25^th^, 75^th^ percentiles) for asymmetric distributions. Categorical variables were presented as counts (proportions). For comparisons between two groups (i.e., metastatic *vs.* non-metastatic groups), normally distributed continuous variables were analyzed using *t*-tests and non-normally distributed variables with Mann-Whitney *U* tests, whereas categorical variables were compared *via* chi-square tests or Fisher’s exact tests. For comparisons across three cohorts (training, internal validation, and external validation cohorts), one-way ANOVA or Kruskal-Wallis *H* tests were applied to continuous variables depending on their distribution, and chi-square tests or Fisher’s exact tests were used for categorical variables as appropriate. The classification performance of different models was quantitatively compared using seven metrics: AUROC, AUPR, accuracy, sensitivity, specificity, precision, and F1 score. When comparing the AUROC of multiple-modality models with single-modality models, AUROC for each model was computed at 1000 bootstrap intervals using R (fbroc package) and statistical analyses were performed using Kruskal-Wallis H tests followed by Dunnett’s multiple comparisons tests. To qualitatively compare the classification performance and clinical benefit of different models, four visualization figures—ROC curve, PR curve, calibration curve, and decision curve—were generated. All statistical analyses were conducted using SPSS (version 26.0, https://www.ibm.com/spss) and R (version 4.2.2, https://www.R-project.org). A two-tailed *p* < 0.05 was considered statistically significant. All figures were created using GraphPad Prism 9 (https://www.graphpad.com/), OriginPro 2021 (https://www.originlab.com/), R (version 4.2.2), and Adobe Illustrator 2023 (https://www.adobe.com/products/illustrator.html).

## 3 Results

### 3.1 Clinical characteristics of the patients

The patients’ demographic baseline characteristics were summarized in **Table 1**. There were 273 ccRCC patients (197 men and 76 women), 216 patients in the training and internal validation cohort and 57 patients in the external validation cohort. The incidence of ccRCC metastasis was 33.33% (72 out of 216) and 29.82% (17 out of 57) in the training and testing cohorts, respectively. Detailed clinical variable comparisons across the training, internal validation, and external validation cohorts were provided in **Supplementary Table 4**. Significant differences were observed in variables such as necrosis, capsule presence, smoking, and drinking habits (all *p* < 0.05), suggesting potential heterogeneity among cohorts. These differences were accounted for during model development through standardized feature normalization.

**Table 1:** Patient clinical characteristics.

Variables	Overall (n = 273)	Non-metastasis group (n = 184)	Metastasis group (n = 89)	*p* value
Age (years)	61.00 (53.00, 68.00)	60.00 (52.00, 68.00)	62.00 (54.00, 68.00)	0.124
Sex (n, %)				0.277
• Female	76 (27.84%)	55 (29.89%)	21 (23.60%)	
• Male	197 (72.16%)	129 (70.11%)	68 (76.40%)	
Location (n, %)				0.542
• Left	133 (48.72%)	92 (50.00%)	41 (46.07%)	
• Right	140 (51.28%)	92 (50.00%)	48 (53.93%)	
Maximum diameter (mm)	4.00 (2.50, 6.00)	3.50 (2.10, 5.03)	5.50 (4.00, 8.50)	**0.001**
Arteriovenous thrombosis (n, %)	31 (11.36%)	11 (5.98%)	20 (22.47%)	**0.001**
Necrosis (n, %)	72 (26.37%)	41 (22.28%)	31 (34.83%)	**0.027**
Lobulation (n, %)	23 (8.42%)	17 (9.24%)	6 (6.74%)	0.486
Lymphadenopathy (n, %)	37 (13.55%)	18 (9.78%)	19 (21.35%)	**0.009**
Capsule (n, %)	49 (17.95%)	34 (18.48%)	15 (16.85%)	0.743
Calcification (n, %)	23 (8.42%)	15 (8.15%)	8 (8.99%)	0.816
Hypertension (n, %)	126 (46.15%)	82 (44.57%)	44 (49.44%)	0.449
Diabetes (n, %)	47 (17.22%)	33 (17.94%)	14 (15.73%)	0.651
Smoking (n, %)	83 (30.40%)	54 (29.35%)	29 (32.58%)	0.586
Drinking (n, %)	84 (30.77%)	49 (26.63%)	35 (39.33%)	**0.033**
Pain (n, %)	48 (17.58%)	30 (16.30%)	18 (20.22%)	0.425
Urination habits (n, %)	35 (12.82%)	20 (10.87%)	15 (16.85%)	0.166
Hematuria (n, %)	42 (15.38%)	23 (12.50%)	19 (21.35%)	0.058
Height (cm)	170.00 (162.00, 172.00)	169.50 (161.75, 172.00)	170.00 (163.00, 173.00)	0.574
Weight (kg)	70.00 (62.00, 80.00)	70.00 (63.00, 78.00)	69.00 (61.00, 80.00)	0.719
Immunoglobulin	26.20 (23.90, 29.20)	25.95 (23.90, 28.52)	26.60 (23.10, 30.60)	0.244
Blood glucose	5.28 (4.83, 6.30)	5.29 (4.82, 6.36)	5.23 (4.92, 6.09)	0.623
Uric acid	314.00 (269.00, 378.00)	304.50 (268.75, 365.75)	314.00 (274.00, 385.00)	0.417
Creatinine	78.00 (64.00, 92.00)	73.00 (63.00, 86.25)	85.00 (72.00, 105.00)	**0.001**
White blood cells	6.44 (5.27, 8.10)	6.36 (5.23, 7.97)	6.52 (5.40, 8.25)	0.273
Neutrophils	4.08 (3.22, 5.78)	4.03 (3.11, 5.40)	4.47 (3.37, 7.01)	**0.016**
Lymphocytes	1.51 (1.10, 1.92)	1.60 (1.17, 2.00)	1.34 (0.97, 1.76)	**0.004**
Basophils	0.03 (0.02, 0.04)	0.03 (0.02, 0.04)	0.02 (0.01, 0.03)	**0.009**
Eosinophils	0.09 (0.04, 0.14)	0.09 (0.04, 0.16)	0.08 (0.03, 0.12)	0.086
Monocytes	0.48 (0.39, 0.63)	0.48 (0.37, 0.64)	0.48 (0.40, 0.62)	0.278
Red blood cells	4.52 (3.99, 4.90)	4.51 (4.04, 4.92)	4.52 (3.94, 4.78)	0.320
Hemoglobin	136.00 (119.00,149.00)	137.00 (121.00, 150.00)	133.00 (115.00, 146.00)	0.216
Mean corpuscular volume	90.00 (86.50, 93.00)	90.20 (86.70, 93.05)	89.50 (85.60, 93.00)	0.433
Platelets	223.00 (180.00,268.00)	224.00 (177.00, 274.50)	220.00 (182.00, 262.00)	0.867
Urinary white blood cells	3.30 (1.40, 13.30)	3.15 (1.30, 13.05)	3.30 (1.90, 15.20)	0.344
Urinary red blood cells	5.90 (1.90, 17.20)	6.00 (1.75, 17.90)	5.80 (2.10, 15.00)	0.938
Number of epithelial cells	1.80 (0.80, 5.00)	1.85 (0.60, 5.18)	1.60 (1.00, 4.90)	0.898

A series of clinical features were compared between the metastatic and non-metastatic groups using Mann-Whitney *U* tests or chi-square tests. A two-tailed *p* value < 0.05 was considered significant difference (highlighted in bold).

### 3.2 Classification performance of single-modality radiomics models

Four single-modality models were constructed using selected features from their respective modalities: the CT_Cortical model, CT_Medullary model, CT_Non-enhanced model, and clinical model. As presented in **Supplementary Table 2** and **Supplementary Figure 1**, the number of selected features for these four models was 6, 4, 7, and 4, respectively. Notably, four clinical features - maximum diameter, creatinine, necrosis, and vascular invasion - were identified as independent risk factors for metastasis in the ccRCC, and were incorporated into the development of the clinical model. The distribution of the maximum diameter of lesions in the training and testing datasets was illustrated in **Supplementary Figure 2**.

**Figure 1:**
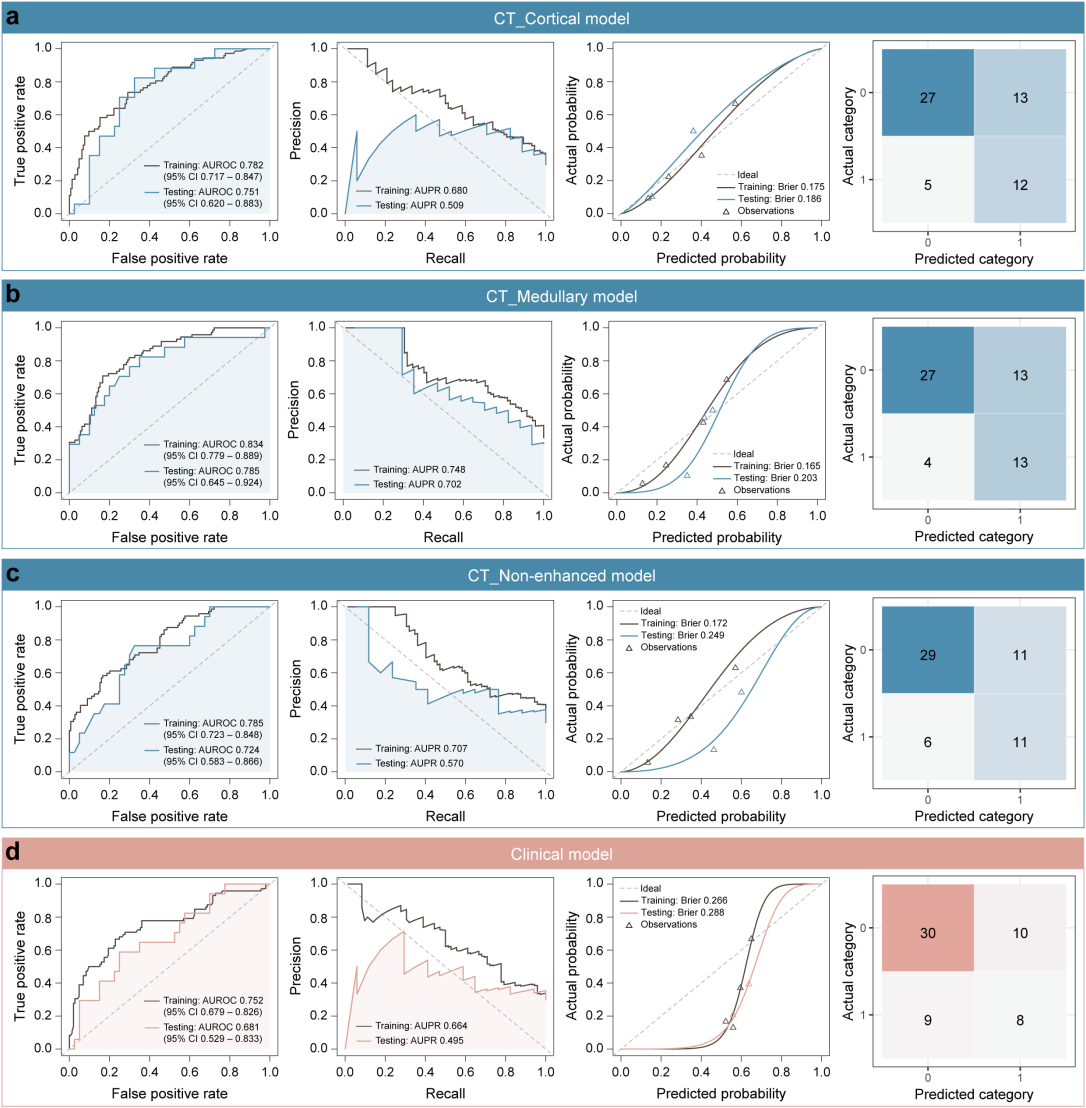
Classification performance of four single-modality models. **(a)** CT_Cortical model, **(b)** CT_Medullary model, **(c)** CT_Non-enhanced model, and **(d)** clinical model. Column 1: Receiver operating characteristic (ROC) curves displayed true positive rate (Y-axis) versus false positive rate (X-axis), with a dashed diagonal representing random classification performance. Column 2: Precision-recall (PR) curves illustrated precision (positive predictive value, Y-axis) against recall (sensitivity, X-axis). Column 3: Calibration curves compared binned predicted probabilities (X-axis) against observed event frequencies (Y-axis), aligned to a perfect-calibration dashed diagonal. Column 4: Confusion matrices used color gradients to show classification outcomes, with larger numbers being darker.

The classification performance of these single-modality models was illustrated in **Figure 1**. In the training dataset, the AUROC values for the single-modality radiomics models based on cortical enhanced, medullary enhanced, and non-enhanced phase images were 0.782 (95% confidence interval [CI]: 0.717-0.847), 0.834 (95% CI: 0.779-0.889) and 0.785 (95% CI: 0.723-0.848), respectively. Correspondingly, in the independent testing dataset, the AUROC values were 0.751 (95% CI: 0.620-0.883), 0.785 (95% CI: 0.645-0.924), and 0.724 (95% CI: 0.583-0.866). Notably, the CT_Medullary model demonstrated relatively superior prediction performance. Decision curve analysis further revealed that both the CT_Cortical and CT_Medullary models provided clinical net benefits across threshold probabilities in both training and testing datasets (**Supplementary Figure 3**). Moreover, the clinical model had an AUROC of 0.752 (95% CI: 0.679-0.826) in the training dataset and 0.681 (95% CI: 0.529-0.833) in the testing dataset. Similar trends were also observed in the PR curves and corresponding AUPR values, with the CT_Medullary model achieving the highest AUPR value. Additionally, calibration curves were employed to assess how well a classification model’s predicted probabilities corresponded to actual outcomes, with a lower Brier score indicating a more accurate model. Furthermore, five other metrics were calculated from the confusion matrices (**Table 2**). Evidently, the CT_Medullary model had the best predictive performance among the four single-modality models, with an accuracy of 0.702 and a sensitivity of 0.765 in the testing dataset.

**Table 2:** Classification performance of six models in the training and testing datasets.

Model	AUROC (95% CI)	AUPR	ACC	F1 score	SEN	SPE	PRE
Training dataset							
Single-modality models							
• CT_Cortical	0.782 (0.717 – 0.847)	0.680	0.625	0.589	0.806	0.535	0.464
• CT_Medullary	0.834 (0.779 – 0.889)	0.748	0.792	0.694	0.708	0.833	0.680
• CT_Non-enhanced	0.785 (0.723 – 0.848)	0.707	0.759	0.500	0.361	0.958	0.812
• Clinical	0.752 (0.679 – 0.826)	0.664	0.769	0.590	0.500	0.903	0.720
Multi-modality models							
• Multi-phased CT	0.837 (0.782 – 0.892)	0.757	0.769	0.662	0.681	0.812	0.645
• Combined	0.969 (0.950 – 0.988)	0.951	0.889	0.844	0.903	0.882	0.793
Testing dataset							
Single-modality models							
• CT_Cortical	0.751 (0.620 – 0.883)	0.509	0.684	0.571	0.706	0.675	0.480
• CT_Medullary	0.785 (0.645 – 0.924)	0.702	0.702	0.605	0.765	0.675	0.500
• CT_Non-enhanced	0.724 (0.583 – 0.866)	0.570	0.702	0.564	0.647	0.725	0.500
• Clinical	0.681 (0.529 – 0.833)	0.495	0.667	0.457	0.471	0.750	0.444
Multi-modality models							
• Multi-phased CT	0.812 (0.680 – 0.943)	0.701	0.789	0.684	0.765	0.800	0.619
• Combined	0.824 (0.704 – 0.944)	0.702	0.772	0.649	0.706	0.800	0.600

Seven quantitative metrics were calculated, i.e., the area under the receiver operating characteristic curve (AUROC) with 95% confidence interval (CI), area under the precision-recall curve (AUPR), accuracy (ACC), F1 score, sensitivity (SEN), specificity (SPE), and precision (PRE).

### 3.3 Model interpretability with SHAP

**Figure 2:**
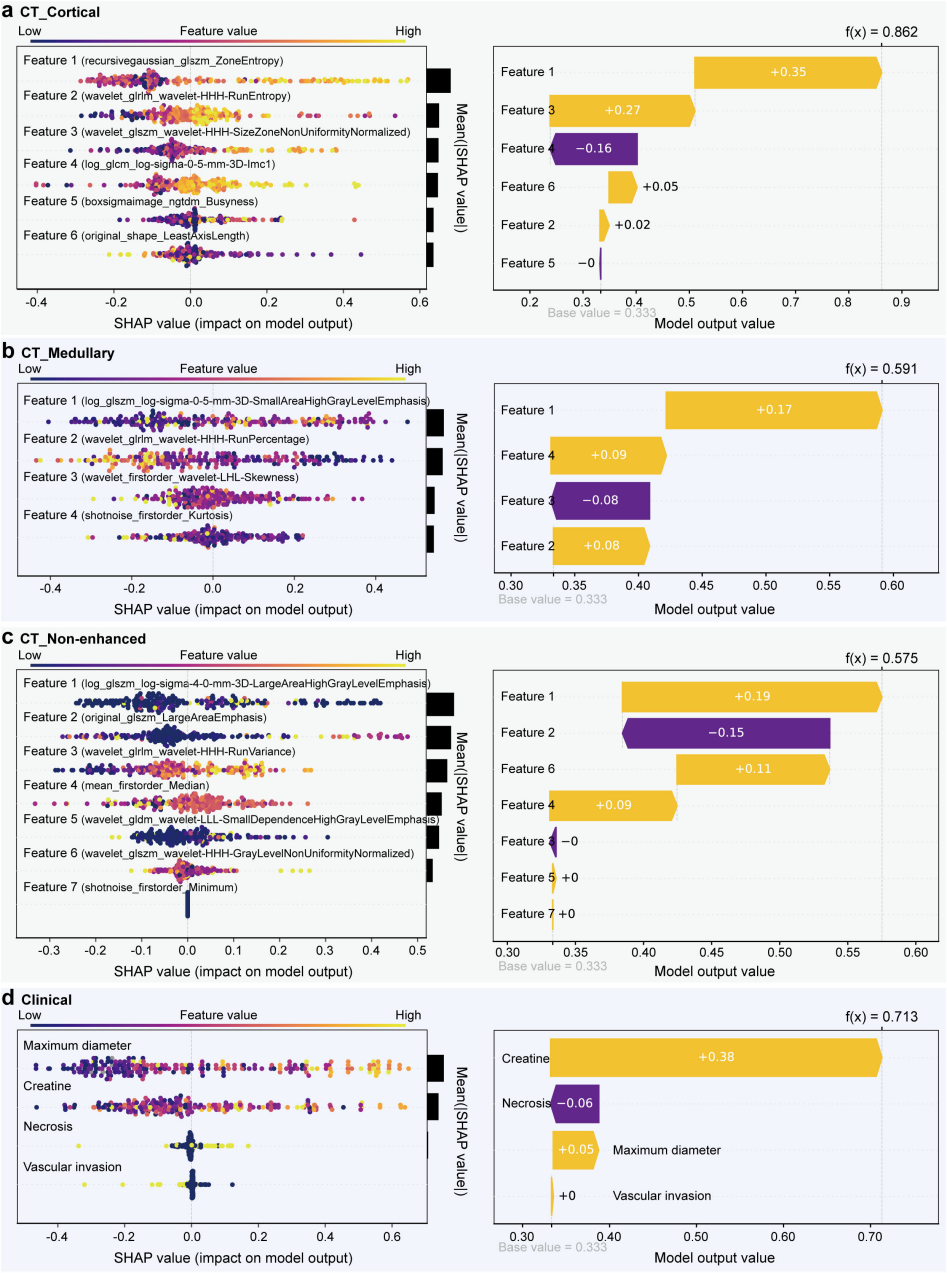
SHapley Additive exPlanations (SHAP) analysis of four single-modality models for clear cell renal cell carcinoma (ccRCC) metastasis prediction: **(a)** CT_Cortical model, **(b)** CT_Medullary model, **(c)** CT_Non-enhanced model, and **(d)** clinical model. Left panel: Summary plots demonstrating directional feature impacts, where horizontal axis SHAP values quantified metastasis risk contribution (rightward = risk-increasing, leftward = risk-reducing), with vertical ordering reflected global feature importance. Color gradients (purple-to-orange) represented feature values (purple = low, orange = high; e.g., elevated “recursivegaussian_glszm_ZoneEntropy” correlated with increased SHAP impact in CT_Cortical model). The bar charts on the right side of the figure ranked features by mean absolute SHAP values (|SHAP|), where bar length corresponded to cumulative impact magnitude across samples. Right panel: Force plots for a representative metastatic ccRCC testing patient, showing consistent baseline risk (base value) versus model-specific decision trajectories (arrows), with final prediction probabilities (f(x)) differing across models due to modality-specific feature contributions (positive/negative impacts shown in orange/purple).

To improve the model interpretability, the SHAP method was used to calculate the Shapley values of each selected feature for the prediction of every observation in single-modality models (**Figure 2**). Positive SHAP values indicated an increased risk of developing metastasis in ccRCC patients. As shown in **Figures 2a-c** three single-modality radiomics models involved 6, 4, and 7 essential features in the global interpretation. The most contributing feature in classifying metastasis and non-metastasis was “recursivegaussian_glszm_ZoneEntropy” in the CT_Cortical model, “log_glszm_log-sigma-0-5-mm-3D-SmallAreaHighGrayLevelEmphasis” in CT_Medullary model, and “log_glszm_log-sigma-4-0-mm-3D-LargeAreaHighGrayLevelEmphasis” in CT_Non-enhanced model. In the clinical model, “maximum diameter” exhibited the largest mean of absolute SHAP value (**Figure 2d**). This demonstrated the similarity between radiomics and clinical information, both of which emphasized the importance of tumor size. In the individual interpretation, one patient was chosen from the independent testing dataset to demonstrate how the SHAP method could be applied to explain individual model predictions. The SHAP force plot showed each feature’s positive and negative effects on the predictive outcomes in a single case. A predictor’s importance was demonstrated by the size of its arrow, where a larger arrow indicated a more important predictor. The base value represented the primary diagnosis and prediction probability, while f(x) represented its final diagnosis and prediction probability.

### 3.4 Construction and evaluation of multi-modality models

**Figure 3:**
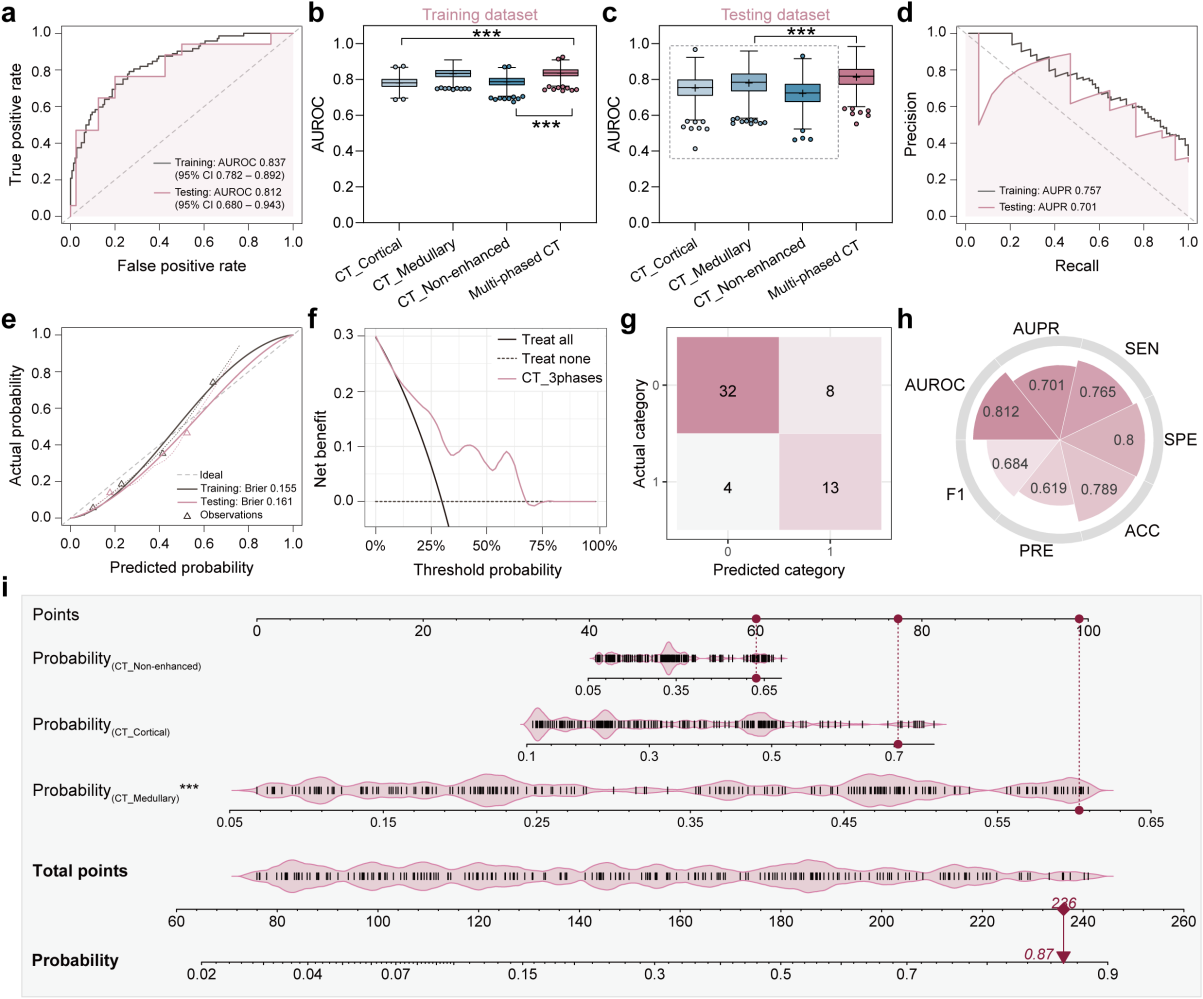
Classification performance and clinical interpretability of the multi-phased CT model. **(a)** Receiver operating characteristic (ROC) curves demonstrated diagnostic accuracy in training (grey line) and testing (pink line) datasets, with Y-axis representing true positive rate and X-axis representing false positive rate. **(b, c)** Area under the ROC curve (AUROC) comparison analyses among four models (CT_Cortical, CT_Medullary, CT_Non-enhanced, multi-phased CT) in training **(b)** and testing **(c)** datasets, where asterisks (***) indicated statistical significance (*p* < 0.001). **(d)** Precision-recall (PR) curves quantified positive predictive value (precision, Y-axis) versus sensitivity (recall, X-axis). **(e)** Calibration curves assessed agreement between predicted probabilities (X-axis) and observed metastasis frequencies (Y-axis), with dashed diagonal denoting perfect calibration. **(f)** Decision curve analysis evaluated clinical net benefit (Y-axis) across threshold probabilities (X-axis) in the testing dataset. **(g)** Confusion matrix used to visualize true *vs.* predicted classifications in the testing dataset. **(h)** Radar plot showed quantitative metrics in the testing dataset including AUROC, area under the PR curve (AUPR), sensitivity (SEN), specificity (SPE), accuracy (ACC), precision (PRE), and F1 score. **(i)** Nomogram integrated predicted probabilities from three single-phase CT models, where vertical red lines marked sample-specific scores for a representative patient, and total points translated to metastasis probability (bottom axis).

Considering the unique information provided by different imaging modalities, multimodal data can harness complementary information to enhance predictive performance. Inspired by this concept, multi-modality fusion models were constructed by integrating the predictive probabilities derived from multiple single-modality models to boost performance. Initially, the Multi-phased CT model was developed by integrating the predicted probabilities from three radiomics models. The model achieved an AUROC of 0.837 (95% CI: 0.782-0.892) in the training dataset and 0.812 (95% CI: 0.680-0.943) in the testing dataset. As illustrated in **Figure 3**, it demonstrated superior performance compared to any individual single-phase model. The enhanced classification performance may be attributed to the fact that three-phase CT images provide a comprehensive view of the lesion information, enabling a more robust prediction of metastasis. Additionally, to better understanding the decision-making process of the model, a nomogram was constructed based on the three probabilities (**Figure 3i**). For a given patient, every variable corresponded to a point, and the total point corresponded to the probability of the metastasis.

**Figure 4:**
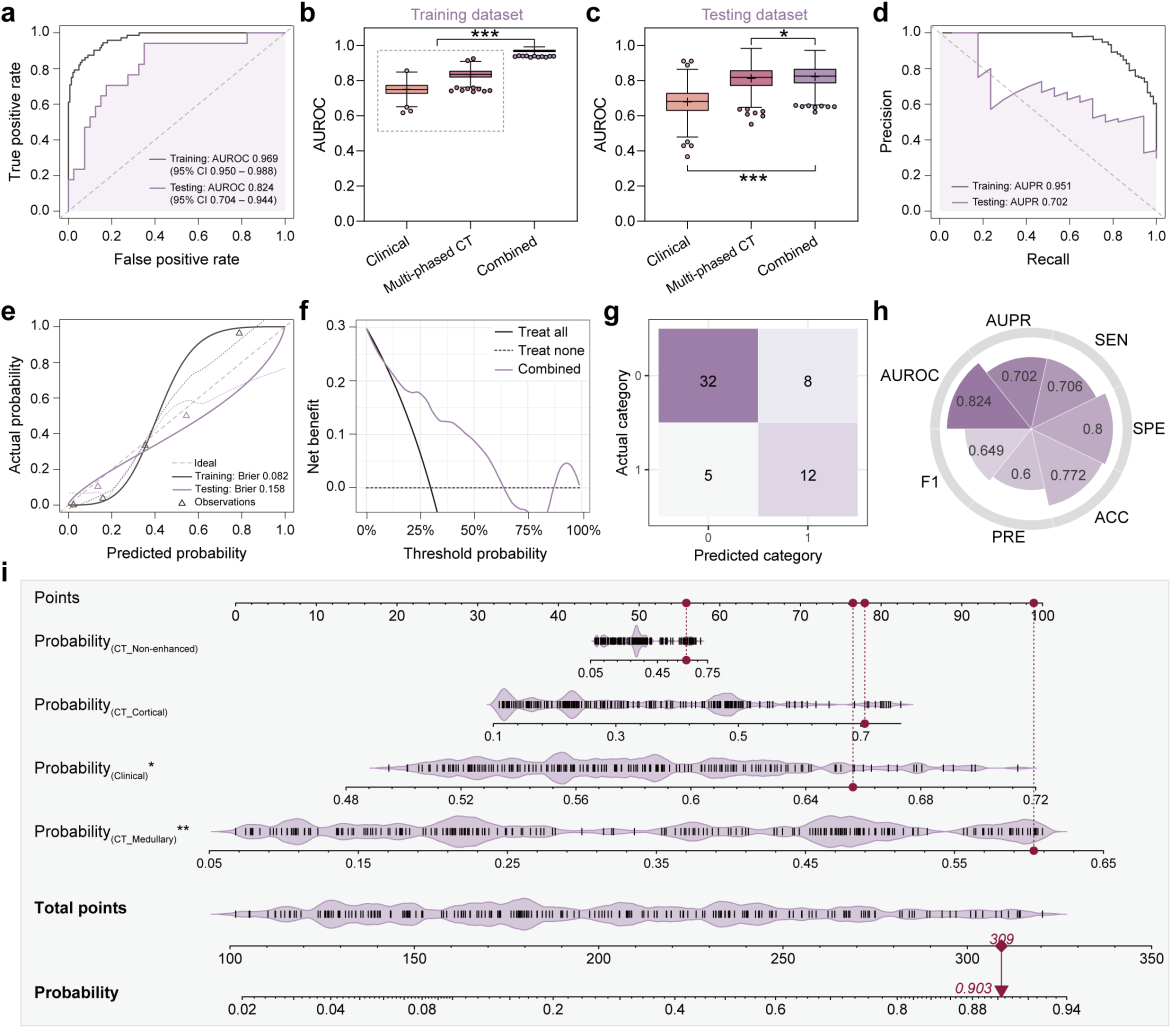
Classification performance and clinical interpretability of the combined model (multi-phased CT + clinical). **(a)** Receiver operating characteristic (ROC) curves demonstrated diagnostic accuracy in training (grey line) and testing (purple line) datasets, with Y-axis representing true positive rate and X-axis representing false positive rate. **(b, c)** Area under the ROC curve (AUROC) comparison analyses among three models (clinical, multi-phased CT, and combined) in training **(b)** and testing **(c)** datasets, where asterisks indicated statistical significance (*p < 0.05, ***p < 0.001). **(d)** Precision-recall (PR) curves quantified positive predictive value (precision, Y-axis) versus sensitivity (recall, X-axis). **(e)** Calibration curves assessed agreement between predicted probabilities (X-axis) and observed metastasis frequencies (Y-axis), with dashed diagonal denoting perfect calibration. **(f)** Decision curve analysis evaluated clinical net benefit (Y-axis) across threshold probabilities (X-axis) in the testing dataset. **(g)** Confusion matrix used to visualize true *vs.* predicted classifications in the testing dataset. **(h)** Radar plot showed quantitative metrics in the testing dataset including AUROC, area under the PR curve (AUPR), sensitivity (SEN), specificity (SPE), accuracy (ACC), precision (PRE), and F1 score. **(i)** Nomogram integrated predicted probabilities from four single-modality models, where vertical red lines marked sample-specific scores for a representative patient, and total points translated to metastasis probability (bottom axis).

To further investigate the complementary ability of radiomics with clinical information, a combined model was constructed by integrating predicted probabilities of the multi-phased CT model with the clinical model. This model achieved an AUROC of 0.969 (95% CI: 0.950-0.988) in the training dataset and 0.824 (95% CI: 0.704-0.944) in the testing dataset **Figure 4**). Pairwise DeLong’s tests were performed to statistically validate its superiority over other models. In the training dataset, the combined model showed significantly higher AUROC values compared to all single-modality models and the multi-phase CT model (all *p* < 0.001). In the testing dataset, while its performance differences against single-modality models were less pronounced, it still demonstrated statistical superiority over the clinical model (*p* < 0.05). These results, visualized as a comparison heatmap in **Supplementary Figure 4** indicated that the three-phase CT radiomics model and the clinical model provide complementary insights into lesion information from various perspectives, thereby enhancing diagnostic performance. Additionally, a nomogram was created that integrated predicted probabilities from four single-modality models, which can help to clarify the decision-making process of the model (**Figure 4i**).

## 4 Discussion

The findings of this study suggest that the unimodal radiomics model, which relies solely on CT image features, and the clinical prediction model, which is based on clinical-radiological features, exhibit limited efficacy in predicting the metastatic risk in patients with ccRCC. However, the integration of radiomics features with clinical data has been demonstrated to significantly enhance the predictive performance. This improvement is mechanistically supported by SHAP (SHapley Additive exPlanations) analysis, which revealed that radiomics features related to intratumoral heterogeneity synergized with clinical factors like tumor size and creatinine levels to drive model predictions. The dominance of radiomics features in SHAP global interpretations underscores their ability to quantify subtle tumor microenvironment characteristics, such as necrosis and hypoxia, which are not fully captured by clinical variables alone. This finding offers valuable insights for the development of personalized treatment strategies for ccRCC patients in clinical practice. Compared to prior studies focusing on single-phase CT or clinical models alone (11–13, 22), our multimodal fusion approach achieved superior performance (testing AUROC: 0.824 vs. 0.68–0.816 in existing literature (22–24)), demonstrating the unique advantage of leveraging complementary information from multiphase CT and clinical-pathological factors.

The observed variation in AUC values across imaging modalities likely reflects distinct pathophysiological insights captured during different contrast phases. For instance, the CT_Medullary model outperformed cortical and non-enhanced models (testing AUROC: 0.785 vs. 0.751/0.724), potentially attributable to its enhanced sensitivity to vascular invasion patterns and hypoxia-induced necrosis during the medullary phase—a period when contrast washout highlights tumor microvascular heterogeneity. This aligns with our SHAP analysis identifying medullary-phase features as key contributors, which may quantify focal necrosis clusters associated with aggressive phenotypes.

Ficarra et al. (25) demonstrated that tumor necrosis, as assessed by the Mayo Clinic Staging, Size, Grade, and Necrosis (SSIGN) scoring system, serves as a significant prognostic factor in the clinical management of ccRCC patients. This finding aligns with our study. SHAP dependence plots further elucidated that radiomics signatures linked to necrosis (e.g., high gray-level emphasis features in medullary-phase CT) exhibited threshold effects, mirroring the biological transition to aggressive phenotypes when necrosis exceeds critical levels. Previous research has established that necrosis occurs when tumor cells exhibit higher metabolic activity relative to angiogenesis levels, resulting in inadequate oxygen and nutrient supply (26). Our investigation revealed that tumor size significantly influences the risk of developing distant metastasis in RCC patients, with larger tumors being more likely to develop such metastasis. SHAP analysis quantified this relationship, showing a sharp increase in metastatic risk contribution for tumors exceeding 3 cm—a threshold consistent with Zastrow et al.’s observations (27). These results are consistent with Hutterer et al.’s (28) study, which developed a nomogram to predict RCC distant metastasis and identified tumor size as a critical risk factor.

The association between intravascular tumor thrombus formation and metastatic risk corroborates previous findings. SHAP local explanations highlighted cases where thrombus-related radiomics features overrode contradictory clinical variables, emphasizing the model’s ability to prioritize imaging biomarkers in context-specific scenarios. Additionally, our study indicated that elevated creatinine levels serve as an independent risk factor associated with metastasis. The near-linear positive correlation between creatinine SHAP values and metastatic risk aligns with its role as a marker of renal dysfunction, which may promote systemic metabolic dysregulation conducive to tumor dissemination (26). The clinical prediction model was developed using the three identified independent risk factors. In both the training and validation datasets, the AUC values were 0.752 (95% CI: 0.679 to 0.826) and 0.681 (95% CI: 0.529 to 0.833), respectively.

Capitanio et al. (23) constructed a predictive model for LNM in kidney cancer, achieving an accuracy of 86.9%. Marconi et al. (24) created prognostic models for survival rates in patients with distant metastases, reporting AUROC values of 0.68 (95% CI: 0.62-0.74) for the preoperative assessment and 0.73 (95% CI: 0.68-0.78) for the postoperative assessment. Bai et al. (22) used MRI images to develop a radiomics nomogram for predicting outcomes in patients with distant metastasis, achieving an AUROC value of 0.816 in the external validation cohort. The AUROC of the multimodal fusion model was 0.969 (95% confidence interval [CI]: 0.950 to 0.988) and 0.824 (95% CI: 0.704 to 0.944) for the training and test sets, respectively. Our multimodal fusion model outperformed these benchmarks (AUROC: 0.969 in training, 0.824 in testing), with SHAP analysis providing critical transparency: it demonstrated how clinical factors (e.g., tumor size) contextualize radiomics patterns (e.g., texture entropy), resolving discrepancies seen in single-modality models. This finding suggests that the integration of multiple sources of information enhances the predictive capability of the model.

Notably, while Bai et al. (22) achieved comparable performance using MRI-based radiomics (external AUROC: 0.816), our CT-based multimodal model offers distinct practical advantages. Firstly, CT remains the first-line imaging modality for ccRCC staging globally, ensuring broader clinical applicability. Secondly, The integration of multiphase CT captures dynamic contrast kinetics, providing insights into tumor angiogenesis and interstitial pressure gradients that MRI cannot replicate. Lastly, Our SHAP-driven nomogram enhances interpretability compared to “black-box” models in prior studies, enabling clinicians to weigh imaging vs. clinical factors case-specifically.

Although the results of this study are encouraging, it is crucial to acknowledge its limitations. Firstly, the retrospective nature of the study may introduce selection bias, potentially compromising the accuracy of the prediction model. Therefore, prospective trials are necessary to validate these findings. Secondly, while all patients underwent CT imaging using contrast agents, it remains unclear whether radiomics features extracted from CT images vary based on different contrast agents and if such variations affect the performance of the final model. Thirdly, although SHAP provided interpretability, causality between specific radiomics features and metastatic pathways remains hypothetical; future studies integrating molecular profiling with SHAP-driven hypotheses are needed. Lastly, despite enrolling patients from three hospitals, the sample size is relatively small. Further studies with larger sample sizes are essential to confirm the accuracy and reliability of the model.

In conclusion, the present study introduced a multimodal fusion model that demonstrated robust performance in predicting the risk of metastasis in ccRCC patients. The SHAP framework not only validated the biological plausibility of feature contributions but also bridged the gap between model complexity and clinical interpretability. The utilization of this model by clinicians has the potential to facilitate more informed and precise treatment decisions.

## Data Availability

The original contributions presented in the study are included in the article/**Supplementary Material**. Further inquiries can be directed to the corresponding author.
