# Supplementary material for: Prediction of metastatic risk of renal clear cell carcinoma based on CT radiomics analysis
Source: Front Oncol. 2025 Jun 6;15:1576956. doi: 10.3389/fonc.2025.1576956 (PMC12178895; doi:10.3389/fonc.2025.1576956)
Supplement: Supplementary file 1 [file DataSheet1.docx]

Supplementary Information

1. Supplementary Tables

All data were collected from three hospitals, as Shandong University Qilu Hospital, Jinan Campus (Hospital A), Shandong University Qilu Hospital, Qingdao Campus (Hospital B), and Changzhou No. 2 People's Hospital (Hospital C)

**Supplementary Table 1. Scanning parameters for renal CT examination.**

| Parameter | Hospital A | Hospital B | Hospital C |
| --- | --- | --- | --- |
| Contrast agent | Ultravist 300, Omnipaque 300 | Ultravist 300, Iopamidol | Omnipaque 300 |
| Injection rate of contrast agent | 2.8 mL/s, 3 mL/s | 2.8 mL/s, 3 mL/s | 3 mL/s |
| Tube current | Automatic modulation | Automatic modulation | Automatic modulation |
| Tube voltage | 100~120 kV | 100~120 kV | 100~120 kV |
| Slice thickness | 5 mm, 1 mm | 5 mm, 1 mm | 1 mm |
| Matrix | 512 × 512 | 512 × 512 | 512 × 512 |
| Convolution kernel | Br40, 130f | Br40, 130f | Br40 |

**Supplementary Table 2. Feature selection and model development protocols for clear cell renal cell carcinoma (ccRCC) metastasis prediction models.** Abbreviations: LASSO (least absolute shrinkage and selection operator), mRMR (minimum redundancy maximum relevance), RF (random forest), DT (decision tree), PLSDA (partial least squares discriminant analysis), LR (logistic regression).

| Model | No. of input features | Feature selection method | No. of selected features | Preprocessor | Classifier |  |
| --- | --- | --- | --- | --- | --- | --- |
| Single-modality models | | | | | | |
| CT_Cortical | 2264 | *F*-test (*P* < 0.05),  LASSO (α = 0.08) | 6 | L2 normalization | RF |  |
| CT_Medullary | 2264 | mRMR | 4 | z-scaler | RF |  |
| CT_Non-enhanced | 2264 | mRMR | 7 | Max_abs_scaler | DT |  |
| Clinical | 42 | Univariate logistic regression (*P* < 0.05), mRMR | 4 | Quantile transformer | PLSDA |  |
| Multi-modality models | | | | | | |
| Multi-phased CT | 3 probabilities | - | 3 | Quantile transformer | LR |  |
| Combined (i.e, Clinical + Multi-phased CT) | 2 probabilities | - | 2 | Quantile transformer | Bagging DT |  |

**Supplementary Table 3. Classifiers and hyperparameters for constructing 6 models.** Abbreviations: RF (random forest), DT (decision tree), PLSDA (partial least squares discriminant analysis), LR (logistic regression).

| Model | No. of selected features | Classifier | Hyperparameters |
| --- | --- | --- | --- |
| Single-modality models | | | |
| CT_Cortical | 6 | RF | No class weight, Gini criterion, max depth = 2, min samples per leaf = 1, min samples per split = 2, number of base estimators = 100 |
| CT_Medullary | 4 | RF | No class weight, Gini criterion, max depth = 2, min samples per leaf = 1, min samples per split = 2, number of base estimators = 100 |
| CT_Non-enhanced | 7 | DT | No class weight, Gini criterion, max depth = 4, min samples per leaf = 1, min samples per split = 2, best splitter |
| Clinical | 4 | PLSDA | Maximum number of iteration steps = 500, number of components = 2 |
| Multi-modality models | | | |
| Multi-phased CT | 3 | LR | No class weight, regularization parameter C = 1.0, penalty parameter of l2, tolerance = 0.0001 |
| Combined | 2 | Bagging DT | Max depth = 4, max features = 1, max samples = 1, number of estimators = 100 |

**(added) Supplementary Table 4. Comparison of clinical variables across training, internal validation, and external validation cohorts.** A series of clinical features were compared among three cohorts using Kruskal-Wallis *H* tests or chi-square tests. A two-tailed *p* value < 0.05 was considered significant difference.

| Variables | Training cohort (n = 175) | Internal validation cohort (n = 41) | External validation cohort (n = 57) | *p* value |
| --- | --- | --- | --- | --- |
| Age (years) | 60.00 (52.00, 66.00) | 61.00 (53.00, 68.00) | 63.00 (55.00, 69.00) | 0.176 |
| Sex (n, %) |  |  |  | 0.232 |
| - Female | 48 (27.43%) | 8 (19.51%) | 20 (35.09%) |  |
| - Male | 127 (72.57%) | 33 (80.49%) | 37 (64.91%) |  |
| Location (n, %) |  |  |  | 0.172 |
| - Left | 81 (46.29%) | 18 (43.90%) | 34 (59.65%) |  |
| - Right | 94 (53.71%) | 23 (56.10%) | 23 (40.35%) |  |
| Maximum diameter (mm) | 4.00 (2.45, 6.25) | 4.80 (2.00, 8.50) | 4.00 (3.00, 5.00) | 0.522 |
| Arteriovenous thrombosis (n, %) | 17 (9.71%) | 10 (24.39%) | 4 (7.02%) | **0.015** |
| Necrosis (n, %) | 24 (13.71%) | 6 (14.63%) | 42 (73.68%) | **< 0.001** |
| Lobulation (n, %) | 9 (5.14%) | 3 (7.32%) | 11 (19.30%) | **0.004** |
| Lymphadenopathy (n, %) | 25 (14.29%) | 11 (26.83%) | 1 (1.75%) | **0.001** |
| Capsule (n, %) | 1 (0.57%) | 1 (2.44%) | 47 (82.46%) | **< 0.001** |
| Calcification (n, %) | 10 (5.71%) | 4 (9.76%) | 9 (15.79%) | 0.056 |
| Hypertension (n, %) | 77 (44.00%) | 19 (46.34%) | 30 (52.63%) | 0.525 |
| Diabetes (n, %) | 33 (18.86%) | 7 (17.07%) | 7 (12.28%) | 0.521 |
| Smoking (n, %) | 60 (34.29%) | 14 (34.15%) | 9 (15.79%) | **0.026** |
| Drinking (n, %) | 67 (38.29%) | 16 (39.02%) | 1 (1.75%) | **< 0.001** |
| Pain (n, %) | 32 (18.29%) | 6 (14.63%) | 10 (17.54%) | 0.858 |
| Urination habits (n, %) | 27 (15.43%) | 6 (14.63%) | 2 (3.51%) | 0.061 |
| Hematuria (n, %) | 30 (17.14%) | 9 (21.95%) | 3 (5.26%) | **0.044** |
| Height (cm) | 170.00 (162.00, 172.00) | 170.00 (161.00, 174.00) | 168.00 (160.00, 170.00) | 0.192 |
| Weight (kg) | 70.00 (62.50, 79.50) | 68.00 (62.00, 80.00) | 70.00 (61.00, 76.00) | 0.972 |
| Immunoglobulin | 25.80 (23.40, 29.10) | 26.60 (23.90, 29.20) | 26.50 (25.30, 29.30) | 0.312 |
| Blood glucose | 5.15 (4.81, 5.85) | 5.14 (4.80, 6.27) | 5.77 (5.26, 7.13) | **< 0.001** |
| Uric acid | 313.00 (270.50, 349.50) | 323.00 (286.00, 391.00) | 342.10 (249.00, 404.20) | 0.251 |
| Creatinine | 75.00 (64.00, 87.50) | 78.00 (67.00, 89.00) | 83.30 (68.80, 101.60) | 0.079 |
| White blood cells | 6.21 (5.22, 7.68) | 6.09 (5.18, 7.32) | 7.55 (5.96, 9.98) | **0.001** |
| Neutrophils | 3.85 (3.15, 5.16) | 4.07 (3.17, 5.60) | 4.81 (3.99, 8.25) | **0.001** |
| Lymphocytes | 1.51 (1.17, 1.93) | 1.48 (1.03, 1.87) | 1.41 (0.97, 1.94) | 0.537 |
| Basophils | 0.03 (0.02, 0.04) | 0.03 (0.02, 0.04) | 0.01 (0.01, 0.02) | **< 0.001** |
| Eosinophils | 0.09 (0.05, 0.16) | 0.09 (0.04, 0.11) | 0.06 (0.03, 0.13) | 0.145 |
| Monocytes | 0.48 (0.39, 0.61) | 0.46 (0.35, 0.61) | 0.49 (0.40, 0.70) | 0.661 |
| Red blood cells | 4.57 (4.03, 4.99) | 4.52 (4.04, 4.71) | 4.33 (3.91, 4.78) | 0.126 |
| Hemoglobin | 138.00 (120.50, 150.00) | 137.00 (122.00, 147.00) | 131.00 (114.00, 142.00) | 0.121 |
| Mean corpuscular volume | 90.20 (86.45, 93.25) | 90.10 (86.70, 93.30) | 88.90 (86.70, 92.30) | 0.378 |
| Platelets | 227.00 (184.00, 276.50) | 224.00 (188.00, 262.00) | 196.00 (160.00, 242.00) | **0.022** |
| Urinary white blood cells | 2.80 (1.40, 10.20) | 2.40 (1.20, 11.10) | 6.00 (3.00, 20.00) | 0.148 |
| Urinary red blood cells | 5.70 (2.10, 15.40) | 5.10 (1.90, 15.20) | 10.00 (0.00, 24.00) | 0.908 |
| Number of epithelial cells | 1.60 (0.80, 5.75) | 2.80 (1.30, 8.00) | 1.00 (0.00, 3.00) | **0.001** |

2. Supplementary Figures


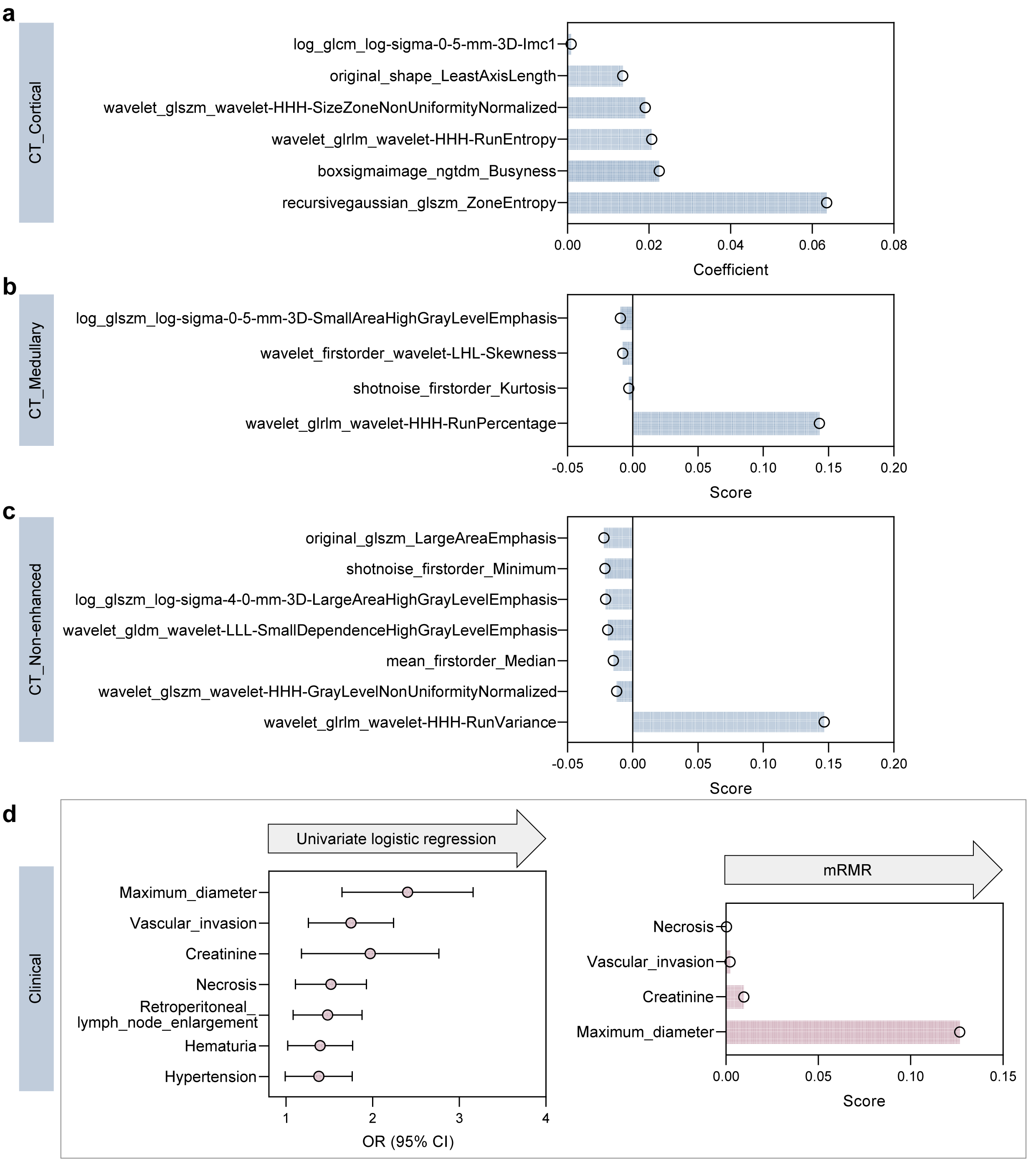


**Supplementary Figure 1. Selected features for constructing single-modality models predicting clear cell renal cell carcinoma (ccRCC) metastasis. (a)** CT_Cortical model: Horizontal axis represented the least absolute shrinkage and selection operator (LASSO) regression coefficients, the magnitude of which indicated the feature contribution weights. CT_Medullary **(b)** and CT_Non-enhanced **(c)** models: Horizontal axis showed the minimum redundancy maximum relevance (mRMR) scores, with higher values indicating stronger feature relevance with metastasis. **(d)** Clinical model: The left panel plotted odds ratios (OR) from univariate logistic regression with horizontal line segments indicating 95% confidence intervals (CI); the right panel displayed mRMR scores for selected features.


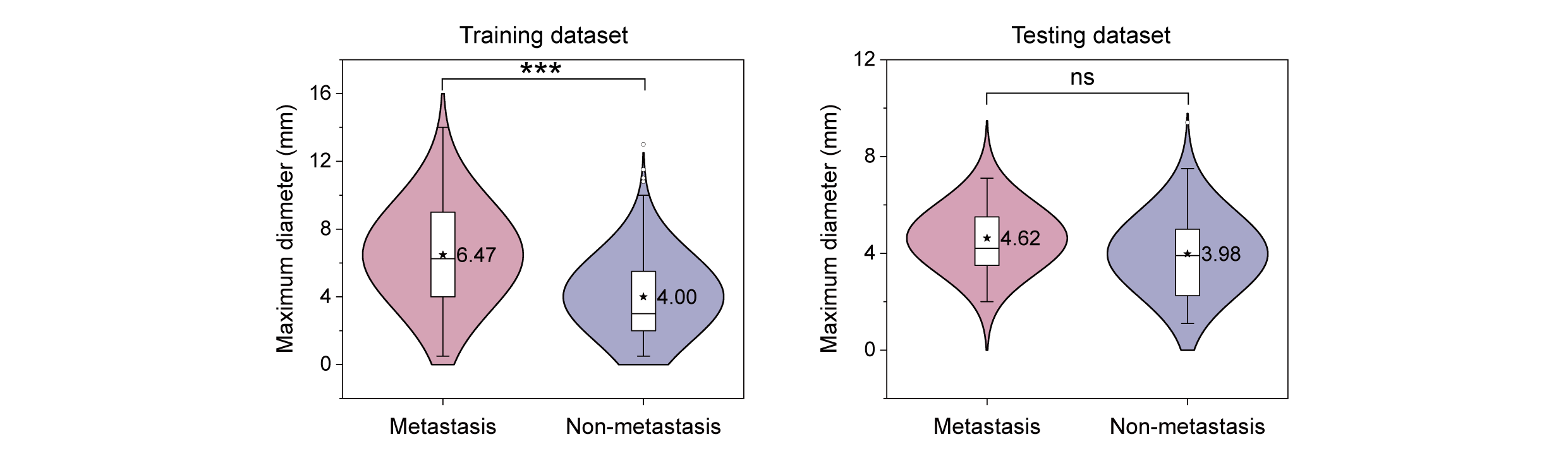


**Supplementary Figure 2. Comparative distribution of lesion maximum diameters between metastatic and non-metastatic groups in the training and testing datasets.** Violin plots (width proportional to probability density) showed kernel-smoothed distributions, with embedded boxplots indicating medians (central line), interquartile ranges (boxes), and 1.5×IQR whiskers. Asterisks denoted statistically significant differences (****p* < 0.001) from Mann-Whitney *U* tests. Numerical annotations reported mean values (mm) for each subgroup. Color coding: Pink = metastatic group, purple = non-metastatic group.


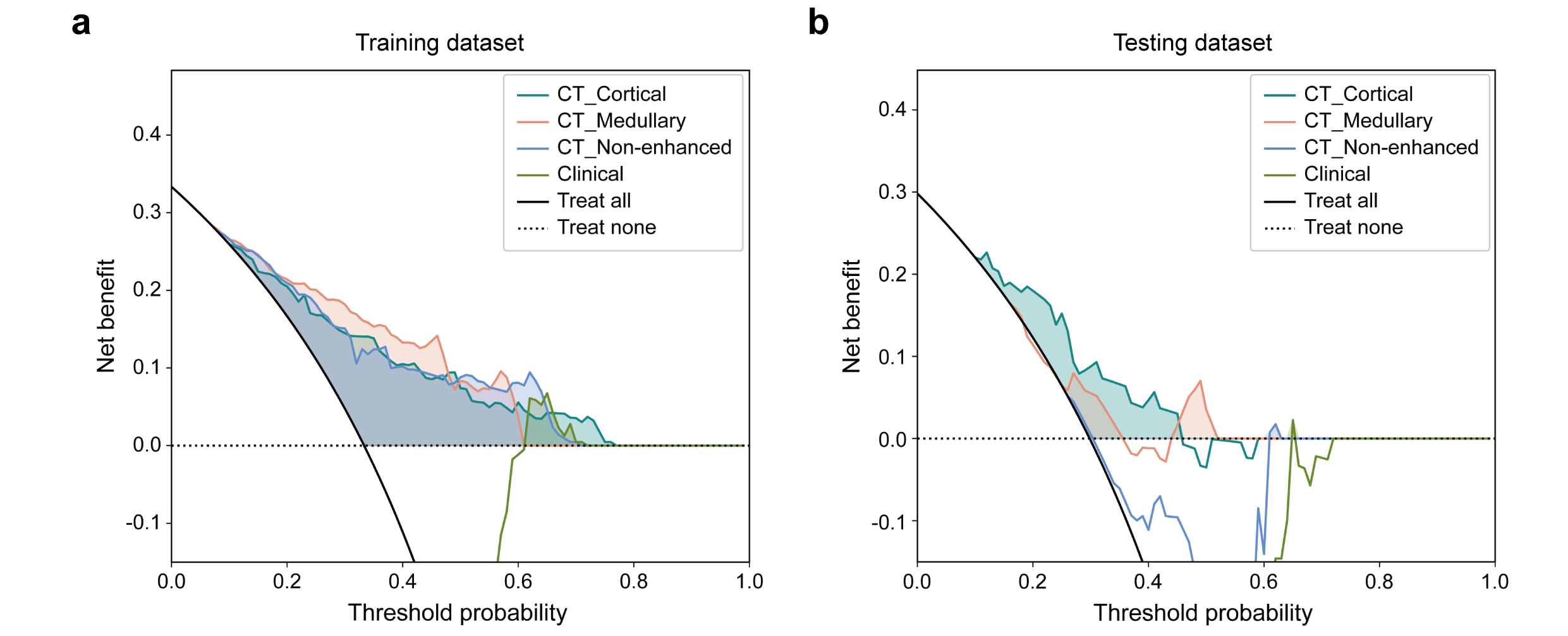


**Supplementary Figure 3. Decision curve analysis (DCA) evaluating clinical utility of four single-modality prediction models for clear cell renal cell carcinoma (ccRCC) metastasis. (a)** Training dataset and **(b)** testing dataset. Net benefit (Y-axis) quantified clinical advantage across threshold probabilities (X-axis). Four model-specific curves were color-coded (CT_Cortical: dark green; CT_Medullary: orange; CT_Non-enhanced: blue; Clinical: light green), compared against “treat all” (black solid line) and “treat none” (black dashed line) reference strategies.


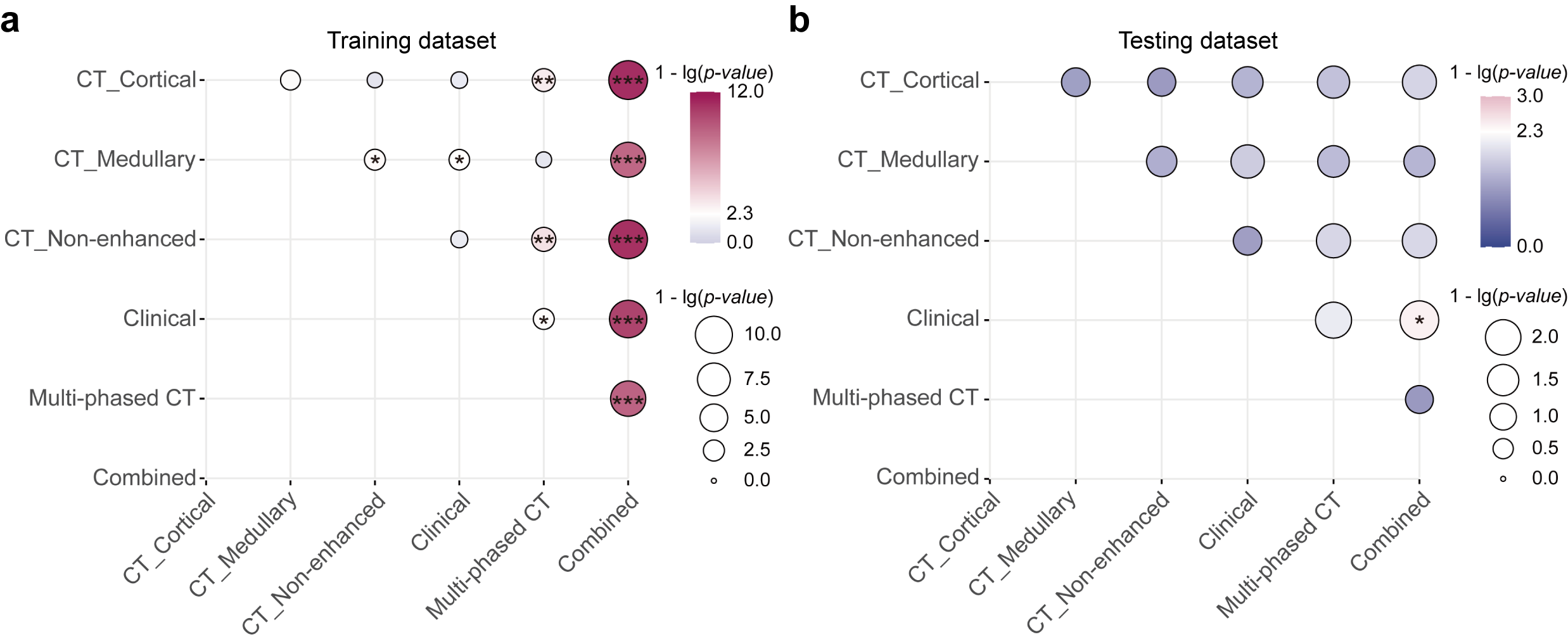


**Supplementary Figure 4. Pairwise comparison of area under the receiver operating characteristic curves (AUROC) values among six models using DeLong’s tests. (a)** Training dataset and **(b)** testing dataset. Rows and columns represented six models, with color intensity indicating statistical significance (red: **p* < 0.05, ***p* < 0.01, ****p* < 0.001; blue: *p* ≥ 0.05) and bubble sizes proportional to 1 − log₁₀(*p-value*), where larger bubbles denoted smaller *p-values* (i.e., stronger evidence of AUROC differences).
